# Cubital tunnel syndrome of the ulnar nerve caused by an epineural ganglion cyst: a case report and review of the literature 

**DOI:** 10.1186/s13256-023-03815-2

**Published:** 2023-03-21

**Authors:** Y. N. Gashi, Mohamed Eltayeb Abdelrahman Naiem

**Affiliations:** Faculty of Medicine, University of Khartoum, Khartoum, Sudan

**Keywords:** Ulnar nerve, Cubital tunnel, Epineural ganglion, Compressive neuropathy

## Abstract

**Background:**

The ulnar nerve has a long and complex anatomical course, originating from the brachial neural plexus in the neck with nerve trunk formation at the posterior neck triangle, and on to the axilla. This intricate anatomical pathway renders the nerve susceptible to compression, direct injury, and traction throughout its course. Compression of the ulnar nerve is the second most common compression neuropathy of the median nerve adjacent to the wrist joint, after carpal tunnel syndrome.

**Case presentation:**

A 45-year-old Sudanese housewife complained of progressive right forearm and hand muscle wasting, pain, and neuropathic symptoms. She was diagnosed with right-sided cubital tunnel syndrome. The diagnosis was derived intraoperatively from a nerve conduction study suggesting the level of conduction block and recommending decompression. Magnetic resonance imaging was not done preoperatively due to financial limitations. An epineural ganglion (15 × 20 mm^2^) compressing and flattening the ulnar nerve was diagnosed intraoperatively. Surgical decompression of the ulnar nerve and removal of the epineural ganglion achieved a remarkable postoperative result and pleasing outcome.

**Conclusion:**

Surgical management is the cornerstone of treatment for compressive neuropathy and ranges from simple nerve decompression to complex neurolysis procedures and nerve transposition to adjust the anatomical course of the nerve.

## Introduction

The ulnar nerve has a long and complex anatomical course, originating from the brachial neural plexus in the neck with nerve trunk formation at the posterior neck triangle, and on to the axilla. This complex anatomical pathway renders the nerve susceptible to compression, direct injury, and nerve traction. Compression of the ulnar nerve is the second most common compression neuropathy of the median nerve, after carpal tunnel syndrome at the wrist joint. The patient’s history, comprehensive clinical neuromuscular examination, appropriate imaging, and electrophysiological testing of the nerve can accurately locate the conduction arrest point and the offending pathology level. Surgical management is the cornerstone of treatment for such conditions, ranging from simple decompression of the nerve to complex neurolysis and nerve transposition, changing the anatomical course of the nerve.

## Case presentation

A 45-year-old right-handed Sudanese housewife presented to the orthopedics surgery clinic with progressive right forearm and hand pain, numbness with a tingling sensation around her right elbow, ulnar side of the forearm and hand. She reported right-hand weakness and decreased grip strength during daily activity. Her signs and complaints had persisted for 1 year, with substantial worsening of the symptoms over the last 2 months. The patient had no other related medical history.

**Fig. 1:**
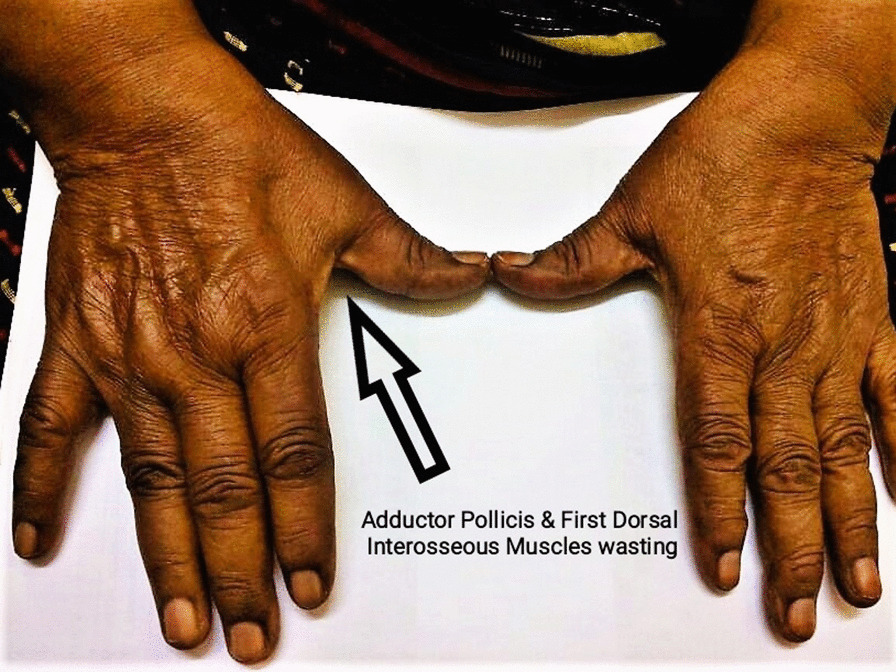
Right-hand muscle wasting compared with the left hand

**Fig. 2:**
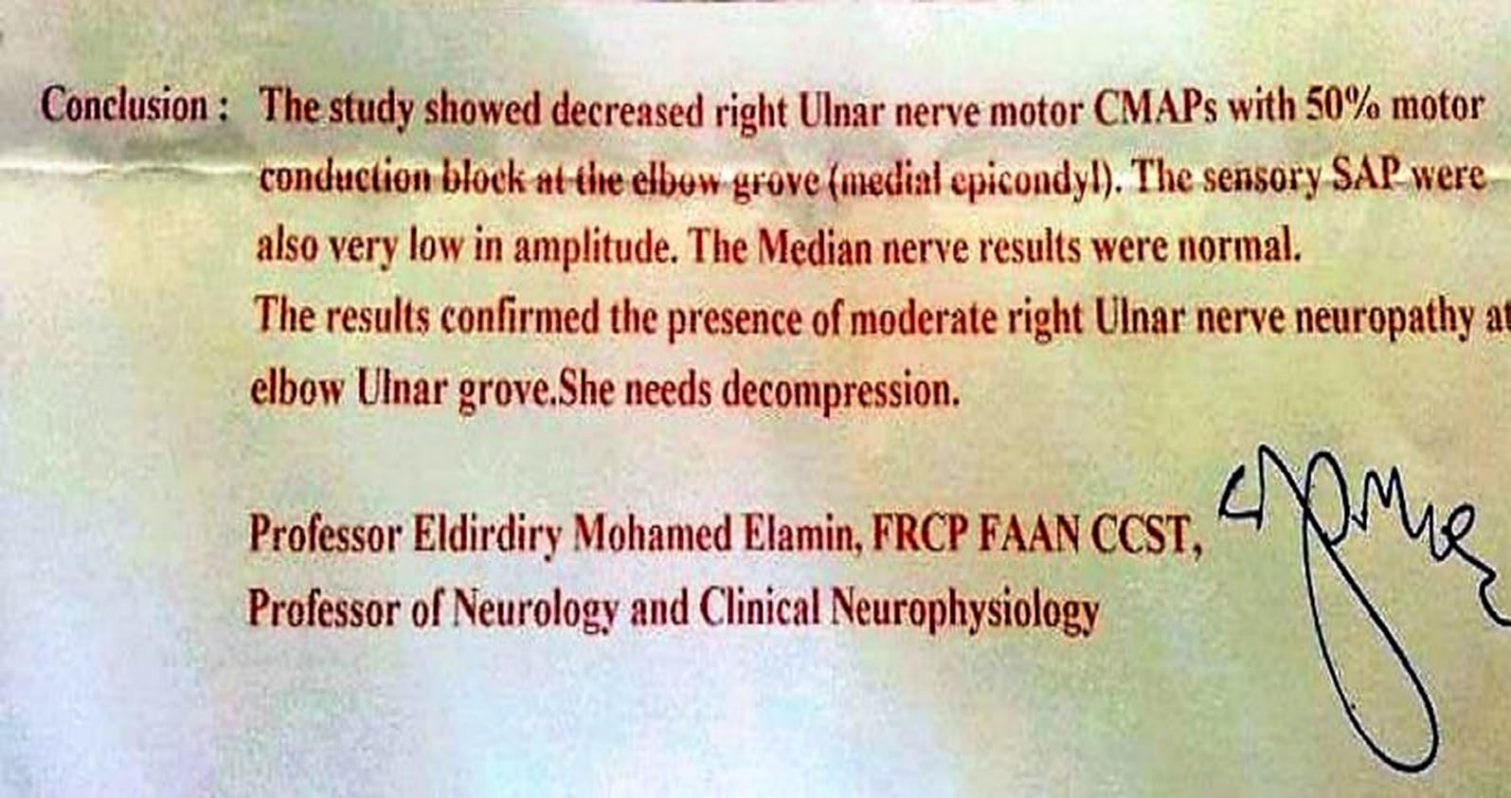
Electrophysiological study report and recommendations

Physical examination revealed a positive Tinel’s sign at the elbow, normal and full range of motion of the elbow, and wasting of the adductor pollicis and first dorsal interosseous muscles (Fig. 1). In addition, the hand grip was weaker on the affected side, with sensation impairment in the ulnar side of the fifth finger and ulnar half of the hand. X-ray of the elbow showed no abnormality. Magnetic resonance imaging (MRI) was not done preoperatively due to financial constraints, but a nerve conduction study demonstrated severe conduction block of both ulnar motor and sensory fibers at the elbow (Fig. 2).

The diagnosis of right ulnar nerve entrapment suggests cubital tunnel syndrome. The patient discussed her condition and was fully informed about her clinical diagnosis supported by the nerve conduction study findings. Surgical exploration was the final decision based upon her preference.

In lateral position and under general anesthesia, we preferred a posterior surgical approach to approach the nerve for direct exploration. The ulnar nerve identified in the cubital tunnel was severely compressed and flattened by a 15 × 20 mm^2^ epineural ganglion.

**Fig. 3:**
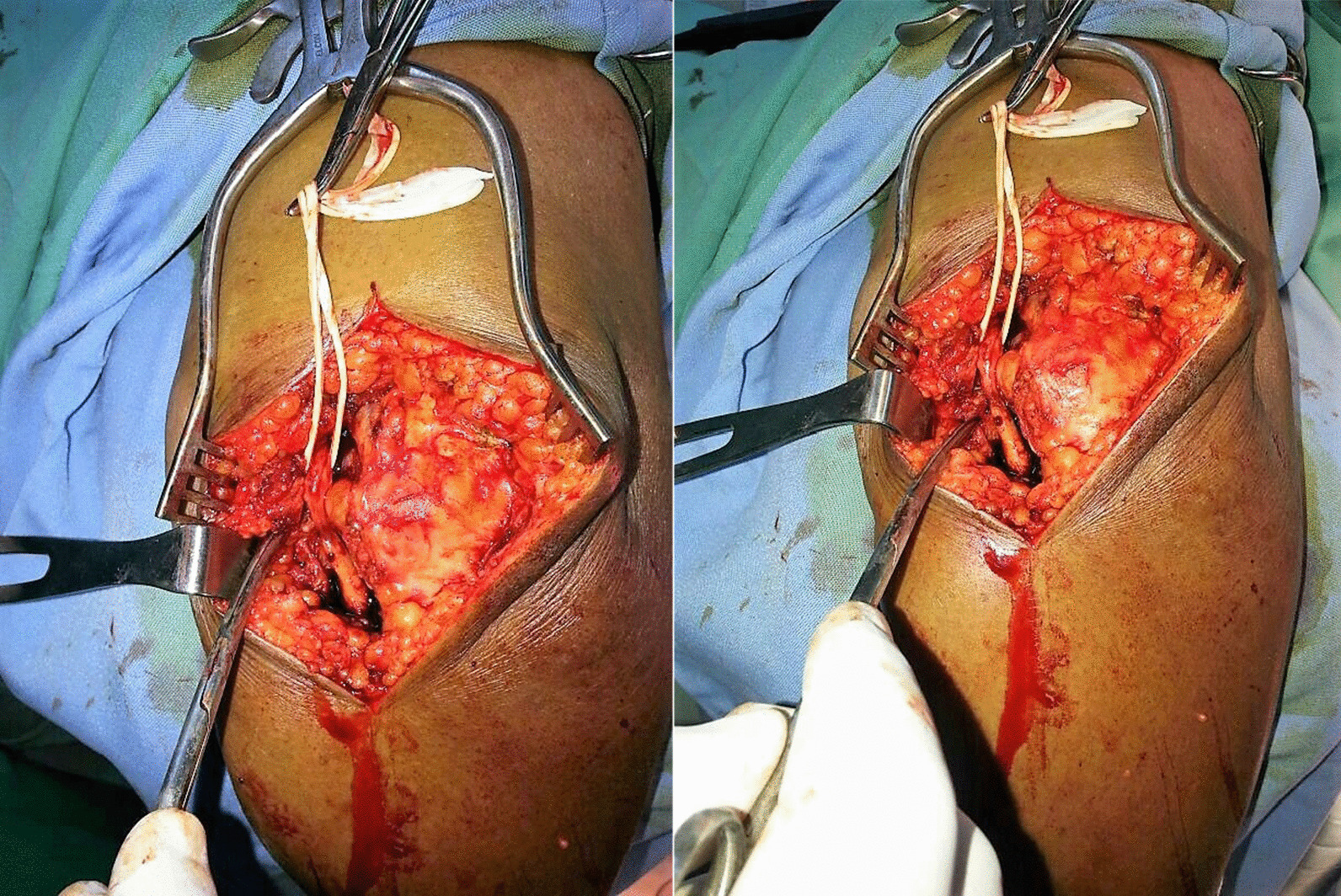
**a**, **b** Intraoperative view of the epineural ganglion

The epineural ganglion was completely excised and the nerve freed (Fig. 3a, b). Histological examination of the cyst confirmed the ganglion cyst diagnosis. A rehabilitation physiotherapy protocol for postoperative recovery followed. Afterward, the patient continued her follow-up for 6 months with complete recovery of sensory and motor function, with significant improvement of hand grip strength and muscle wasting clinically.

## Discussion

Compressive neuropathy affecting the ulnar nerve leads to neuromuscular signs and symptoms at the level of the cubital region and is the second most common after carpal tunnel syndrome [1, 2]. It affects males three to eight times more often than females owing to the nerve’s anatomical course, making it vulnerable to over-employment injury and occupational impairment.

The etiology ranges from physiological compression, which occurs during elbow flexion, to tumors in the tunnel, counting bursae, ganglion cysts, inflammatory conditions affecting the elbow joint, and osteophytes, with an epineural ganglion being one of the rare causes; the overall incidence of ganglion cysts is 8%. Most of them originate from the ulnar–humeral joint [2, 3].

Most of the ganglion cysts causing cubital tunnel syndrome arise from the elbow joint and represent 8% of the overall etiology; the cyst-to-nerve relation can be intrinsic or extrinsic to the neural sheath and epineurium [4]. In our case, the ganglion cyst was confined to the epineural sheath of the ulnar nerve, being a so-called epineural ganglion, with only a few cases reported in literature of epineural ganglions causing ulnar nerve compression in the cubital tunnel [3, 5–12].

In our case, the long history, progressive severity of the symptoms, and physical examination findings directed us toward this diagnosis, in addition to the evident wasting of the adductor pollicis muscle, the first dorsal interosseous muscle, impaired ulnar nerve distribution sensation, in conjugation with the weaker hand grip on the right hand. Unfortunately, a handheld grip strength measuring device is unavailable in our country.

All of these findings mandated an electrophysiological study, which confirmed that the ulnar nerve was compressed at the elbow groove (medial epicondyle) with 50% motor block and very low-amplitude sensory action potentials (SAPS).

The patient’s progression, considering that she is a right-handed housewife and the effect on her daily life activities, besides the inability to do further imaging studies, made surgical exploration of the nerve mandatory. Preoperative confirmation by MRI scan provides an opportunity for simple aspiration of the cyst, either directly or image-guided. Unfortunately, financial limitations and restricted access to MRI rendered the patient unable to have a preoperative MRI scan, which led us to offer surgical exploration and decompression.

The diagnosis of epineural ganglion was made intraoperatively, with simple decompression by careful excision of the compressing ganglion cyst and neurolysis of the nerve. Minimum disruption of the nerve sheath and anatomy of the nerve course was achieved, compared with other reported cases, in which some authors prefer to do an anterior transposition of the nerve after excision of the ganglion.

The patient’s symptoms resolved, her motor and sensory functions recovered, and her hand grip strength improved significantly. Therefore, the outcome is considered excellent compared with the preoperative status [13, 14]. The choice between cubital tunnel syndrome treatment modalities depends on the patient’s complaint, symptoms, duration, progression, physical activity, the effects on daily life, and the pathology causing the syndrome. It can include medical therapy, physiotherapy, and surgical therapy.

Surgical therapy is usually a second-line therapy, but essential if the pathology is surgically treatable, with the most beneficial outcome expected. Decompressing the affected nerve provides the most satisfactory results. Ulnar nerve transposition after decompression and medial epicondylectomy remains a valid option [15].

## Conclusion

Surgical management is the keystone of treatment in such situations, varying from uncomplicated nerve decompression to complex neurolysis and nerve transposition to modify the anatomical course of the nerve.

## Data Availability

The datasets utilized in this article are obtainable upon reasonable request from the corresponding author. All medical data, supporting materials, and images are available upon request.
